# Complete Human Penile Scaffold for Composite Tissue Engineering: Organ Decellularization and Characterization

**DOI:** 10.1038/s41598-019-51794-6

**Published:** 2019-11-08

**Authors:** Yu Tan, Wilmina N. Landford, Matthew Garza, Allister Suarez, Zhengbing Zhou, Devin Coon

**Affiliations:** 1 Department of Plastic & Reconstructive Surgery, Johns Hopkins University School of Medicine, 600 N. Wolfe Street, Baltimore, Maryland 21287 USA; 2 Translational Tissue Engineering Center, Department of Biomedical Engineering, Johns Hopkins University Whiting School of Engineering, Baltimore, Maryland USA; 3 Department of Hand & Microsurgery, Xiangya Hospital of Central South University, 87 Xiangya Road, Changsha, Hunan Province 410008 P.R. China

**Keywords:** Tissue engineering, Translational research

## Abstract

Reconstruction for total penile defects presents unique challenges due to its anatomical and functional complexity. Standard methods suffer from high complication rates and poor functional outcomes. In this work we have developed the first protocol for decellularizing whole-organ human penile specimens for total penile tissue engineering. The use of a hybrid decellularization scheme combining micro-arterial perfusion, urethral catheter perfusion and external diffusion enabled the creation of a full-size scaffold with removal of immunogenic components. Decellularization was complete as assessed by H&E and immunohistochemistry, while quantification of residual DNA showed acceptably low levels (<50 ng/mg). An intact ECM was maintained with histologic architecture preservation on H&E and SEM as well as preservation of key proteins such as collagen-1, laminin and fibronectin and retention of growth factors VEGF (45%), EGF (57%) and TGF-beta1 (42%) on ELISA. Post-decellularization patency of the cavernosal arteries for future use in reseeding was demonstrated. Scaffold biocompatibility was evaluated using human adipose-derived stromal vascular cells. Live/Dead stains showed the scaffold successfully supported cell survival and expansion. Influence on cellular behavior was seen with significantly higher expression of VWF, COL1, SM22 and Desmin as compared to cell monolayer. Preliminary evidence for regional tropism was also seen, with formation of microtubules and increased endothelial marker expression in the cavernosa. This report of successful decellularization of the complete human phallus is an initial step towards developing a tissue engineered human penile scaffold with potential for more successfully restoring cosmetic, urinary and sexual function after complete penile loss.

## Introduction

Complex urogenital and penile defects commonly result from congenital, traumatic, or neoplastic disease as well as in gender confirmation surgeries. Reconstruction for these patients presents unique challenges due to the anatomical and functional complexity of the human phallus^1,2^. Ideal goals for reconstruction include formation of a cosmetically realistic neophallus, functional urethra for voiding, tactile and erogenous sensation, and erectile functionality for penetrative sexual activity. Current approaches to penile reconstruction primarily include pedicled and free microsurgical skin flaps which are limited in their ability to recapitulate the complex architecture of the penis and cause significant donor site morbidity^3,4^. These approaches struggle particularly to reconstruct complex urogenital tissues.

The radial forearm free flap with its relatively hairless skin, accessible innervation, and functional capabilities supportive of neourethral extension and penile prosthesis insertion, has been regarded as the gold standard^4^. However, obtaining an acceptable aesthetic appearance, patent urethra, erogenous sensation, and rigidity for sexual penetration is frequently challenging. Multiple operations are typically necessary to not only repair the underlying deformities but also complete the staged process and can result in significant donor site morbidity, prosthesis extrusion, urethral strictures/fistulas, and inferior aesthetic outcomes^4,5^. Additional unsolved limitations to this procedure are reflective of limitations in recapitulating unique tissues such as the tunica albuginea or urethral epithelium. Furthermore, patients with traumatic penile loss and concomitant extremity injuries, especially wartime injuries, may lack donor sites for conventional reconstruction, necessitating alternative strategies^6^.

Organ allotransplantation continues to remain the primary option for the restoration of complex, multi-functional organs^6^. Our group recently performed the most extensive penile and perineal transplant to date for reconstruction of a wartime penile injury^7^. Successful allogenic penis transplants have the potential to restore form and specialized function. However, patients must take a cocktail of immunosuppressant drugs throughout their lifetime with major risks including infection and cancer. Rejection of whole organ transplants has been reported as high as 65% within the first 10 years^8^. The complications of lifelong immunosuppression and chronic rejection continue to hinder the success and applicability of transplantation. There continues to be a need for reconstructive solutions in the case of complete penile loss from cancer or trauma.

Tissue engineering has provided a growing repertoire of techniques aimed at overcoming the hurdles associated with autologous reconstruction and allotransplantation. Decellularization, in particular, offers the potential to retain the structure and functionality of the native organ, while limiting immunogenic effects for the patient. Decellularized organ scaffolds have been increasingly used in regenerative strategies for tissue and organ replacement by providing the appropriate biomechanical support necessary while setting the groundwork for micro-environmental cues during cell attachment, proliferation, and differentiation with the patient’s own cells^9–12^.

A main challenge of penile reconstruction is its anatomic complexity. Each of the cavernosa contains arteries and a vascular network as well as spongy tissue, encased in tunica to support rigidity, while the corpus spongiosum is filled with spongy tissue, smooth muscle and vessels, surrounding the actual urethra with its specialized lining of pseudostratified columnar epithelium. Despite being mainly composed of smooth muscle and endothelial cells, even engineering one corporal body unit alone remains a challenge. Several studies have evaluated the feasibility of bioengineering pendular penile corporal bodies in a rabbit model, however, no studies have been done using human corporal tissue^13–15^. For clinical applicability, it is necessary to achieve successful decellularization and reseeding where each structure is several times larger than the rabbit, increasing standard diffusion/perfusion challenges in tissue engineering.

In this work we demonstrate our protocol for complete decellularization of human penile specimens to create the first complete penile scaffold, with reduction of immunogenic factors below standard safety thresholds while maintaining extracellular matrix (ECM) architecture and growth factors. Preliminary cytocompatibility of the scaffold is shown by successful re-integration of cells during reseeding. Our data suggests that creation of a penile organ scaffold that retains the necessary architecture for recellularization and translational clinical application is possible.

## Methods

**Figure 1:**
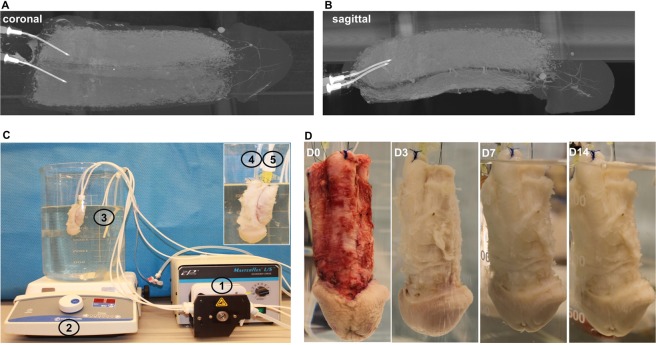
Organ scaffold decellularization system. (**A**,**B**) Micro-CT radiographic images demonstrating the intricate vasculature via perfusion of contrast into the cavernosal arteries. (**C**) Perfusion system consisting of a (1) peristaltic pump, (2) magnetic stirrer plate, and a (3) 4 L glass container. The scaffold has been cannulated with a (4) Foley catheter in the urethra and (5) two angiocatheters in the cavernosal arteries. (**D**) Photographic representation of the decellularization process at 0, 3, 7, and 14 days, respectively.

Composite human penectomy specimens were collected under Johns Hopkins IRB approval after patient consent. All aspects of the studies outlined were performed in accordance with all regulations and guidelines. Eighteen specimens were collected as part of transgender surgery and were from patients aged 18–45. Informed consent was obtained for each sample. Based on prior literature in decellularization of similar tissues^16^, a solution of 1% SDS in DI water was chosen. In initial work, experiments were performed to identify the ideal perfusion rate and time for specimens, which was found to be a rate of 1 ml per minute and duration of 14 days at room temperature (Fig. 1). The degree of decellularization was qualitatively assessed by histology, residual cellular content analysis, and ECM component evaluation to investigate decellularization efficiency and ECM preservation. Urethral sub-structure analysis included the mucosa, submucosa and spongiosum.

Vascular patency before and after was assessed by micro-CT angiography. Lastly, 300 µm slices of the scaffold were reseeded using stromal vascular fraction (SVF) cells to study the cytocompatibility of the decellularized scaffold to support cell attachment and proliferation.

### Specimen cannulation and decellularization protocol

The main artery in each cavernosum was cannulated under a surgical microscope with a 24-gauge intravenous catheter followed by placement of a lasso stitch to hold it in place. The cannula was then used to perfuse the penis tissue with 1% sodium dodecyl sulfate (SDS) and 1% Antibiotic-Antimycotic (Thermo Scientific, Pittsburgh, PA) in deionized water for 2 weeks in a 4 L glass beaker, and the SDS solution was changed every 48 hours. For the final protocol at low-flow rate (1 ml/min) an 18–22 Fr Foley catheter was placed into the urethra and SDS perfusion was also performed through this route. The submerged penectomy specimen underwent slow mechanical agitation (50 rpm) on a magnetic stir plate while being perfused.

### Perfusion pressure characterization

Catheters with pressure sensors (AD instruments, Colorado Springs, CO) were connected to the perfusion tube cannulating the specimen during decellularization. Pressures were monitored and recorded by a LabChart system (AD instruments, Colorado Springs, CO) for 10 minutes for averaging data analysis.

### Stromal vascular fraction (SVF) cell isolation and cell culture

After rinsing human fat tissue with 1x PBS, surgical scissors were used to morselize tissue. Morselized fat tissue was transferred to 25 ml of 1x PBS, supplemented with 0.15% collagenase type 1 (Thermo Scientific, Pittsburgh, PA) and incubated for 45 min with mechanical agitation (250 rpm) at 37 °C. After digestion, the tissue was centrifuged at 600 g for 10 minutes and the supernatant was carefully aspirated. Cell pellets were resuspended in media. Finally, SVFs were seeded in T75 flasks and cultured with Dulbecco’s Modified Eagle Medium (DMEM, 4500 mg/L glucose) (Thermo Scientific, Pittsburgh, PA) containing 10% FBS (Thermo Scientific, Pittsburgh, PA) and 1% Antibiotic-Antimycotic (Thermo Scientific, Pittsburgh, PA).

### Cell seeding on decellularized penis scaffold sections

300 μm thickness slices of decellularized penis scaffold were sectioned using a cryostat (Leica Microsystems, Inc., Exton, PA). Slices were directly deposited into ultra-low attachment petri dishes and sterilized with 1x PBS supplemented with 1% Antibiotic-Antimycotic (Thermo Scientific, Pittsburgh, PA). Before cell seeding, slices were emulsified in medium for 30 min at 37 °C. Slices were semi-dried at room temperature in the cell culture hood for 30 min to adhere to the petri dish surface. SVF cells (1 × 10^6^ cells) were deposited on the slice. Seeded slices were incubated at 37 °C for 30–40 min after cell deposition to allow for adsorption, after which medium was added for long term culture.

### DNA measurement

DNeasy Blood & Tissue Kit (Qiagen, Hilden, Germany) was used to isolate DNA from penile tissue before and after decellularization based on the manufacturer’s protocol. Briefly, 20 mg of dissected tissue pieces were placed in a 1.5 ml microcentrifuge tube with 180 μl of Buffer ATL and 20 μl of proteinase K. After vortexing, the sample was incubated at 56 °C until completely lysed (around 30 min). Then 200 μl of 100% ethanol was added to the mixture and centrifuged in a spin column. After washing twice with Wash Buffer, DNA was eluted with 200 μl Buffer AE. Finally, the DNA concentration (ng/DNA per mg/tissue) was measured with a NanoDrop 1000 (Thermo Scientific, Pittsburgh, PA).

### Enzyme-linked immunosorbent assay (ELISA)

VEGF, EGF and TGF-beta1 in all three different parts (cavernosa, tunica and urethra) of penis tissue were measured with ELISA kits (Cusabio, Houston, TX) based on the manufacturer’s protocol. Briefly, around 100 mg tissue was homogenized in 1 mL PBS and stored overnight at −20 °C. After two freeze-thaw cycles, the homogenates were centrifuged at 5000 g for 15 minutes. The supernatant was then collected in a clean vial as the test sample. First, 100 uL samples and standard controls were added to each well in the antibody-coated plate and incubated for 2 hours at 37 °C in the incubator. After removing the liquid, 100 uL biotin antibody was added and incubated for another 1 hour at 37 °C in the incubator. After triple rinsing with washing buffer, 100 uL horseradish peroxidase avidin was added and incubated for another 1 hour at 37 °C. After five times rinsing with washing buffer, 90 uL TMB substrate was added and incubated for 15 minutes at 37 °C before adding 50 uL of the stop solution. The absorbance values were measured with a spectrometer at 450 nm (BioTek, Winooski, VT). The concentrations of growth factors were calculated based on the standard curve.

### Immunofluorescent staining

After washing (3 times at 5 min) in 1x PBS with 0.1% Triton X-100 (PBST), 100 µl of blocking buffer was added (10% goat serum in 1x PBS) onto sections and incubated in a humidified chamber at room temperature for 1 h. Sections were incubated with appropriately diluted primary antibody: alpha smooth muscle actin (Millipore, Billerica, MA), CD31 (Abcam, Cambridge, UK), collagen-1 (Abcam, Cambridge, UK), laminin (Abcam, Cambridge, UK) and fibronectin (Abcam, Cambridge, UK) overnight at 4 °C. After washing in PBST (3 times at 5 min), tissues were incubated with fluorescent secondary antibodies diluted in PBST for 1 h at ambient temperature. After washing in PBST (3 times at 5 min), nuclei were stained with DAPI (Molecular Probes/Invitrogen, Eugene, OR) diluted in PBST for 15 min at ambient temperature. Following the final washing procedure (PBST, 3 times at 5 min), glass cover slips were added to the slides using Fluoro-Gel (Electron Microscopy Sciences, Hatfield, PA). Immunostained samples were imaged with a LSM510 laser scanning confocal microscope (Zeiss, Oberkochen, Germany).

### Scanning electron microscopy (SEM)

Tissues were fixed for 30 minutes in 10% formalin solution. After dehydration with increasingly concentrated ethanol solutions (50%, 70%, 80%, 95%, 100%), tissues were soaked in the chemical drying agent Hexamethyldisilazane (Sigma-Aldrich, Darmstadt, Germany) for 3 times at 20 min. Samples were incubated in Hexamethyldisilazane overnight and sputter-coating was subsequently performed. The sample was coated with a layer of 10 nm thickness of gold and gadolinium (Denton Vacuum). Finally, images were taken by a field emission scanning electron microscope (Zeiss, Oberkochen, Germany).

### Micro-CT angiography protocol

20 ml Microfil (Flow TEch, Inc., Carver, MA, USA) was perfused into the penis specimen through two arteries located in the cavernosa at room temperature. The samples were fixed over night at 4 °C in 10% buffered formalin phosphate. Lastly, the samples were CT scanned (nanoScan PET/CT Small Animal Imager, Mediso, Hungery).

### Statistical methods

All data were expressed as mean ± standard error of the mean (SEM). One-way ANOVA was used for statistical analysis. Statistical differences between groups from the same experimental set were determined using the Tukey post-hoc test. A p-value < 0.05 was considered significant.

## Results

### Decellularization of whole-organ penile scaffold

The size of the specimens (up to 15 cm in length) rendered simple diffusion-based decellularization approaches clearly non-viable. Perfusion-based decellularization is an effective way to remove cellular components to produce a bioactive extracellular matrix (ECM) scaffold with preservation of the intricate three-dimensional micro-architecture and vascular networks of native tissue^17^. Micro-CT images of specimens showed the cross-connectivity of the blood vessel network and presence of vascular structure extending all the way to the glans (Fig. 1A,B), demonstrating potential for decellularizing the majority of the penis through the two arteries located in the cavernosa.

Assessment of perfusion pressures through the pump was performed to provide insight into flow rate selection. At the initial rate of 4 mL/min (selected as the physiological blood flow rate in deep cavernous arteries^18^), pressures exceeded the apparatus measurement threshold of 500 mm Hg, while after lowering to 1 mL/min pressures decreased to 357 ± 26 mm Hg (p < 0.001). The high flow rate resulted in visible enlargement of the specimen, presumably due to pressurization, while the lower flow rate did not. During erections, blood pressure in penile tissue can reach several hundred mmHg^19^, suggesting that the perfusion pressure associated with a flow of 1 mL/min during decellularization was expected to be viable. Given the small diameter of the corporal arteries (<1 mm) and resulting challenges of increasing flow further from Pouiselle’s law, 1 mL/min was adopted.

Additional cannulation of the urethra with a Foley catheter was added after initial low-flow 1 mL/min experiments to obtain the final protocol, as a simple route to improve decellularization efficiency in the portion of the specimen least perfused by the cavernosal arteries (i.e., the distal, ventral urethra).

The perfusion system (Fig. 1C) applied for penis decellularization consists of a peristaltic pump, a magnetic stirrer plate and a 4 L glass beaker containing 1% SDS in DI water supplemented with 1% antibiotic-antimycotic to prevent sample contamination during multi-week processing.

### Preservation of histological morphology and gross anatomy

**Figure 2:**
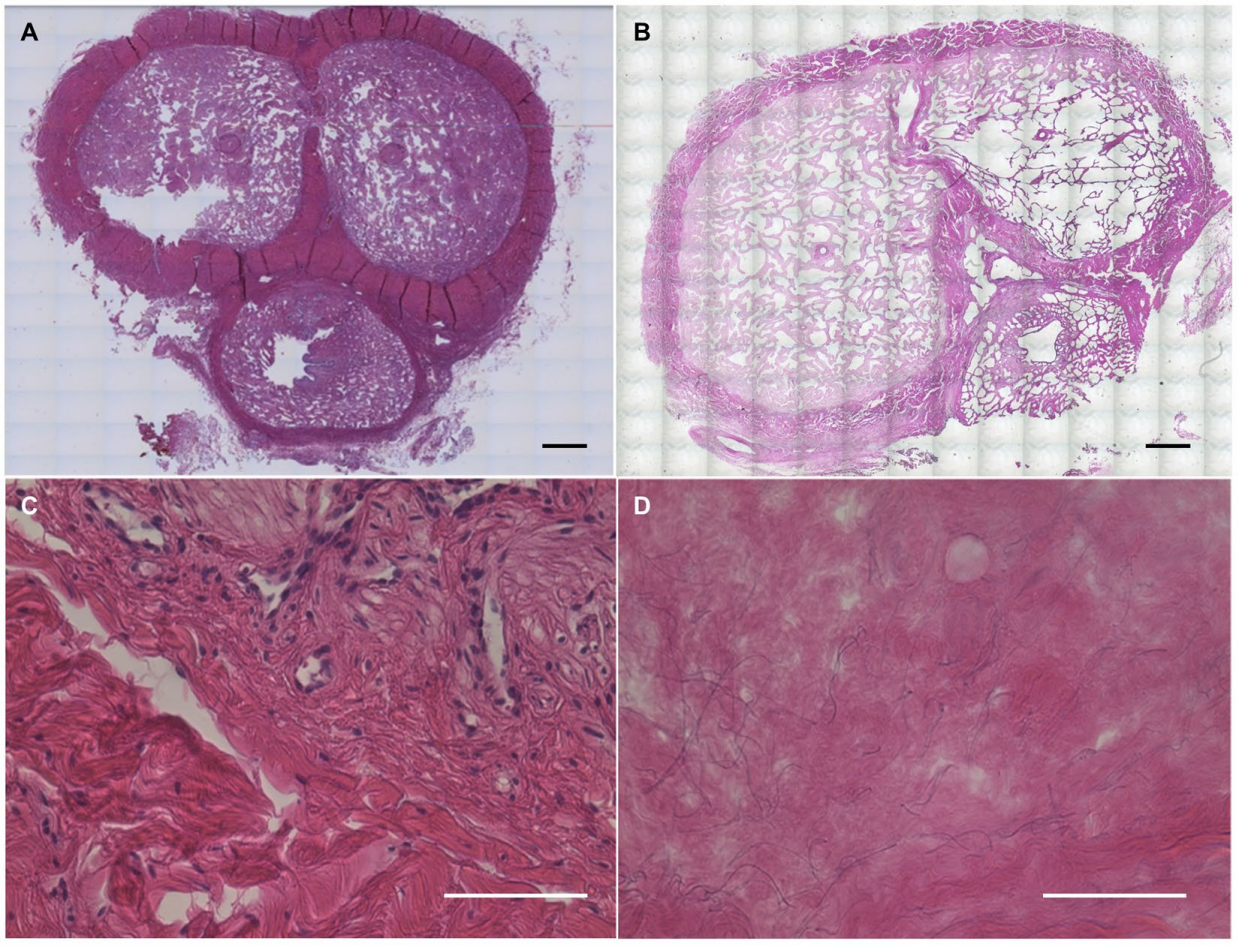
Hematoxylin & Eosin staining for assessment of decellularization. Bright field microscopy of H&E staining for cross section of penis specimen before (**A**,**C**) and after (**B**,**D**) decellularization, demonstrating elimination of all nuclei with preservation of ECM morphology. Scale bar: (A,B) = 3 mm; (C,D) = 100 µm.

Overall progress of decellularization was followed by the macroscopic appearance of penis tissue, which became totally white at day 14 due to progressive removal of cellular components in the tissue (Fig. 1D). Both gross examination and tiled H&E microscopy demonstrated successful preservation of a patent urethral lumen throughout the length of the scaffold (Fig. 2). Maintenance of patent, paired cavernosal arteries for future re-endothelialization was seen (Supp Fig. 1).

### Characterization of decellularization success

**Figure 3:**
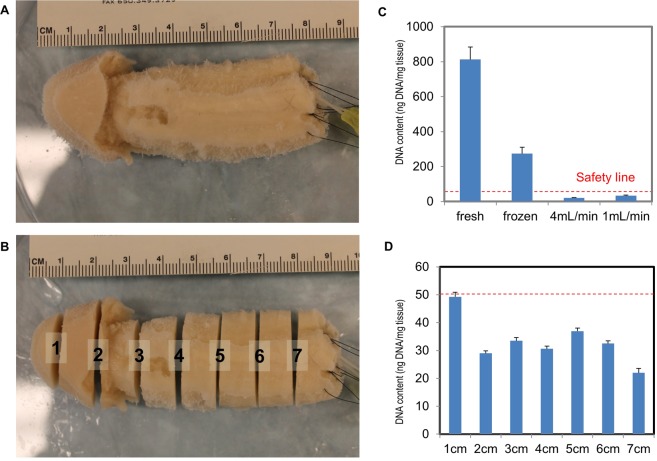
DNA content quantification studies. A decellularized penile scaffold (**A**) before, and (**B**) after sectioning at 1 cm intervals. (**C**) Comparisons of fresh, frozen, and decellularized tissue at 4 mL/min and 1 mL/min perfusion rate for 2 weeks were quantified to evaluate DNA content. A red dotted-line indicates the threshold for an acceptable level of residual DNA which generates minimal to no post-implantation immunoresponse (50 ng DNA/mg tissue). (**D**) Sectioned slices along the organ scaffold are processed for DNA content quantification and to confirm a fully decellularized organ scaffold.

A key criterion for successful tissue decellularization is the level of residual DNA after processing, which correlates with scaffold immunogenicity. A typical safe threshold of DNA level is 50 ng per mg tissue for minimal immunoresponse post-implantation^16^. Our results show the residual DNA content in the decellularized penis with perfusion rate 4 mL and 1 mL per minute is 21.0 ± 2.2 and 33.3 ± 3.1 ng/mg tissue respectively (Fig. 3C), both of which were well within the safety threshold.

It was also necessary to investigate the residual DNA distribution spatially in the decellularized penis, due to its large dimensions. The decellularized penis sample was sectioned at 1 cm intervals (Fig. 3A,B) and DNA content measurement was conducted for each section. The results indicate the residual DNA level of all the sections are within the safe limit (Fig. 3D), though the distal glans part was marginally higher than the other areas due to distance from the cannulated arteries.

**Figure 4:**
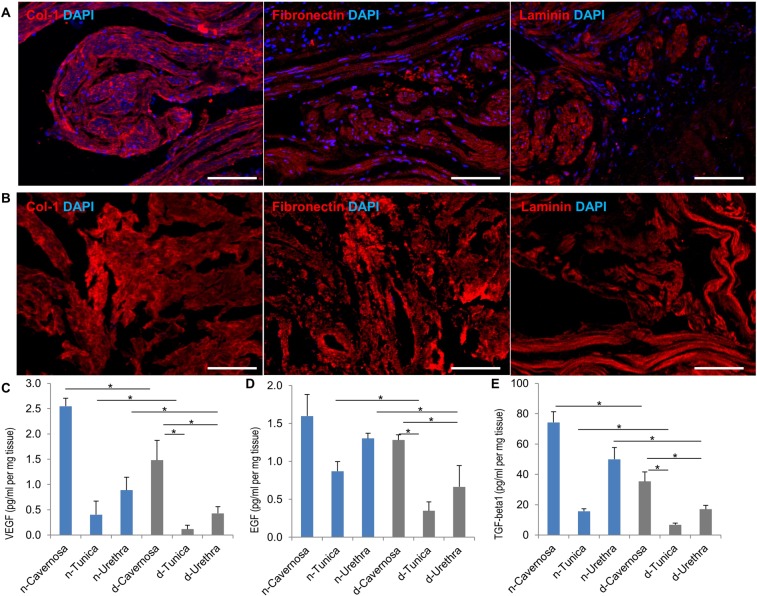
Extracellular matrix proteins assessment. Immunofluorescent staining of ECM proteins (red; Collagen-1, Fibronectin and Laminin) and cell nuclei (blue; DAPI) in cavernosal tissue before (**A**) and after (**B**) decellularization, demonstrating preservation of proteins with elimination of cells. (**C**–**E**) Quantification of vascular endothelial growth factor (VEGF), epidermal growth factor (EGF) and transforming growth factor beta 1 (TGF-beta1) in all three parts (cavernosa, tunica and urethra) of native (n) and decellularized (d) penile tissue respectively. N = 3; *P < 0.05. Scale bar: 100 µm.

The other criterion for successful tissue decellularization is that there should no visible nuclei after processing. The data of H&E staining and DAPI staining before and after decellularization (Figs 2, 4, Supp Figs 1, 2) show that there are no visible or detectable nuclei after decellularization in all parts of the penis (cavernosa, tunica, urethra and glans), which confirmed that our perfusion protocol successfully removed all the cellular components from penis tissue.

### Preservation of scaffold extracellular matrix and biochemical composition

Based on the immunofluorescent staining of three common representative proteins in the ECM (collagen-1, fibronectin and laminin) for cavernosa, tunica and urethra in the penile scaffold (Fig. 4A,B and Supp Fig. 2), there was no significant loss in these critical biochemical components after decellularization. Laminin was much more abundant in the cavernosa than the other parts of penile scaffold while the tunica had minimal expressed levels.

**Figure 5:**
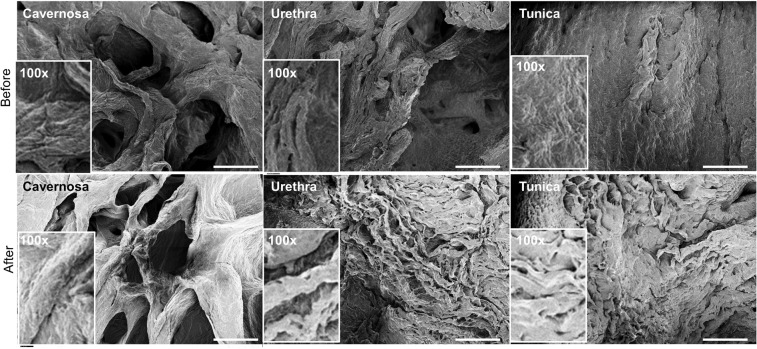
Scanning electron microscopy (SEM) of organ scaffold. SEM images of three key anatomical structures of the penis (cavernosa, tunica albuginea and urethra) before and after decellularization illustrating retention of the unique micro-topography of each. Inset represents high-magnification view (100X). Scale bar: 200 µm.

SEM images reveal similar morphologies before and after decellularization in the cavernosa, tunica and urethra in the scaffold, which indicates that our decellularization protocol retained the unaltered ECM micro-topography, such as more porosity in the cavernosa and urethra parts versus more dense fibrous structures in the tunica (Fig. 5). Major cell types in penile tissue include endothelial cells, smooth muscle cells, urethral epithelium cells and fibroblasts. Thus, changes of growth factors relevant to each cell type (VEGF for endothelial cells, EGF for urethral epithelial cells and TGF-beta1 for smooth muscle cells and fibroblasts) were evaluated by ELISA before and after decellularization. These major cytokines are critically important for reconstruction of penile tissue^20^. Some loss of soluble growth factors is invariably seen with detergent-based decellularization, but Fig. 4C–E shows preservation of the growth factors of VEGF, EGF and TGF-beta1 at 45%, 57% and 42% on average respectively compared with native tissue. The cavernosa consistently had the significantly highest level for all three growth factors. In aggregate, our data suggests that the decellularized penile scaffold retains a similar biochemical composition and topography.

### Preliminary recellularization of penile scaffold

To achieve our ultimate goal (clinical penile reconstruction), recellularization of the scaffold is obviously required. As large numbers of cells will be necessary due to the size of the scaffold, cell lines capable of being obtained in significant quantity from recipients are of primary interest. Therefore, the adherent population of the cells within the stromal vascular fraction (SVFs) were successfully isolated and cultured from human adipose tissue and used for initial reseeding experiments. Phenotype detection by flow cytometry indicated that the majority of cells were adipose-derived stem cells with 98.34% positive for CD105, 93.19% positive for CD90 and 97.03% negative for CD45 (Supp Fig. 3).

**Figure 6:**
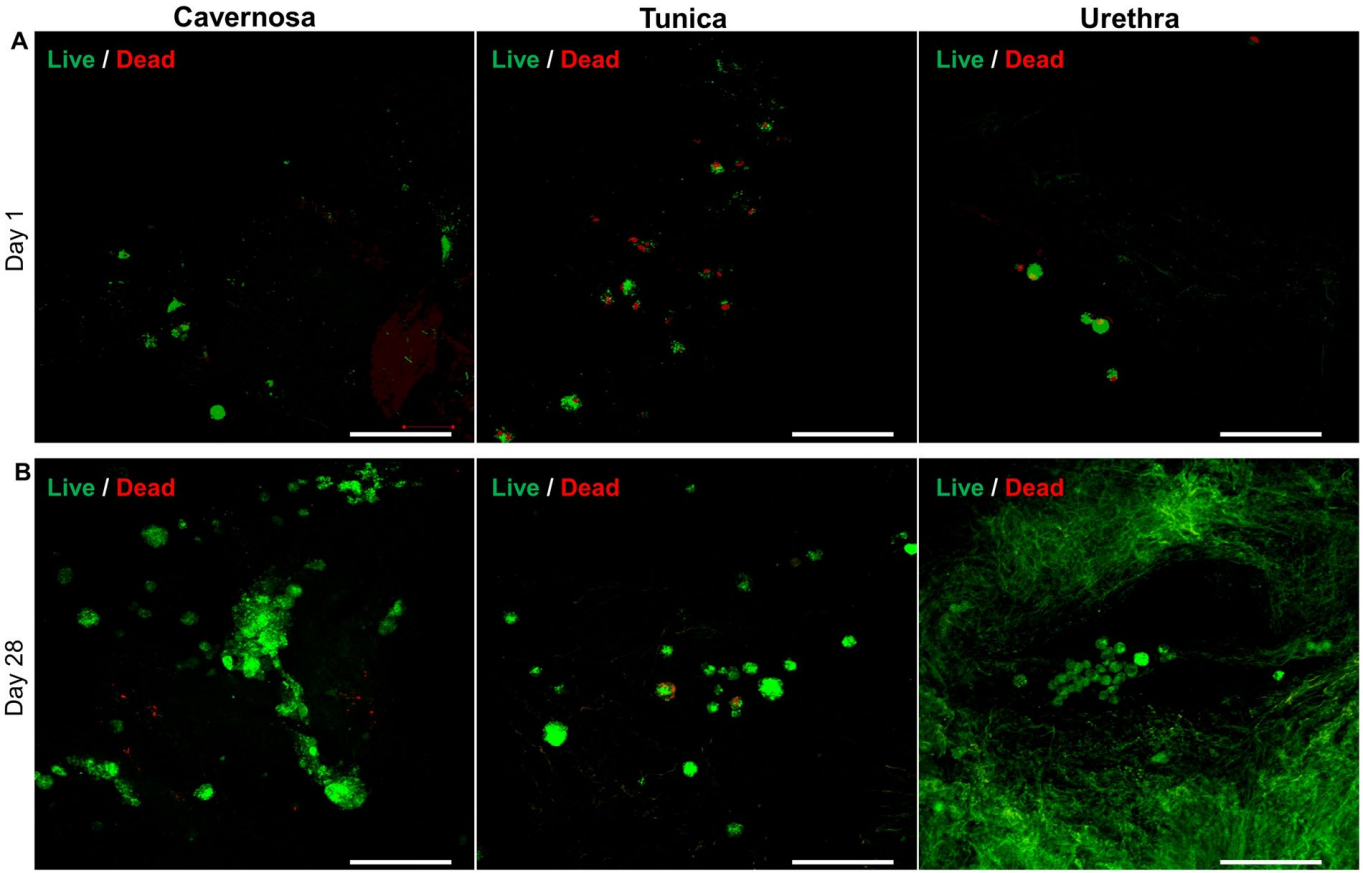
Cell viability assessment after scaffold reseeding with SVFs. Immunofluorescence with live/dead stain (green color indicates live cells and red color indicates dead cells) of SVFs seeded onto decellularized scaffold cross-sections at days 1 (**A**) and 28 (**B**) of urethra, tunica and cavernosa. Scale bar: 100 µm.

In our pilot experiment reseeding the penile scaffold, the cellular adhesion, migration, proliferation, differentiation and organization were examined by seeding SVFs on thin-slices of the scaffold from day 1 to day 28. Live/Dead staining at day 1 post cell seeding indicated that cells attached on the surface quickly with high viability (Fig. 6A).

Higher densities of viable cells in all three parts (cavernosa, tunica and urethra) at day 28 shows that cells could proliferate in the decellularized penile scaffold over time to recapitulate tissue (Fig. 6B). Interestingly, cells formed small clusters in the cavernosa and urethra parts, with tube-like structure formation observed in the cavernosa region.

### Characterization of cellular behavior after reseeding

**Figure 7:**
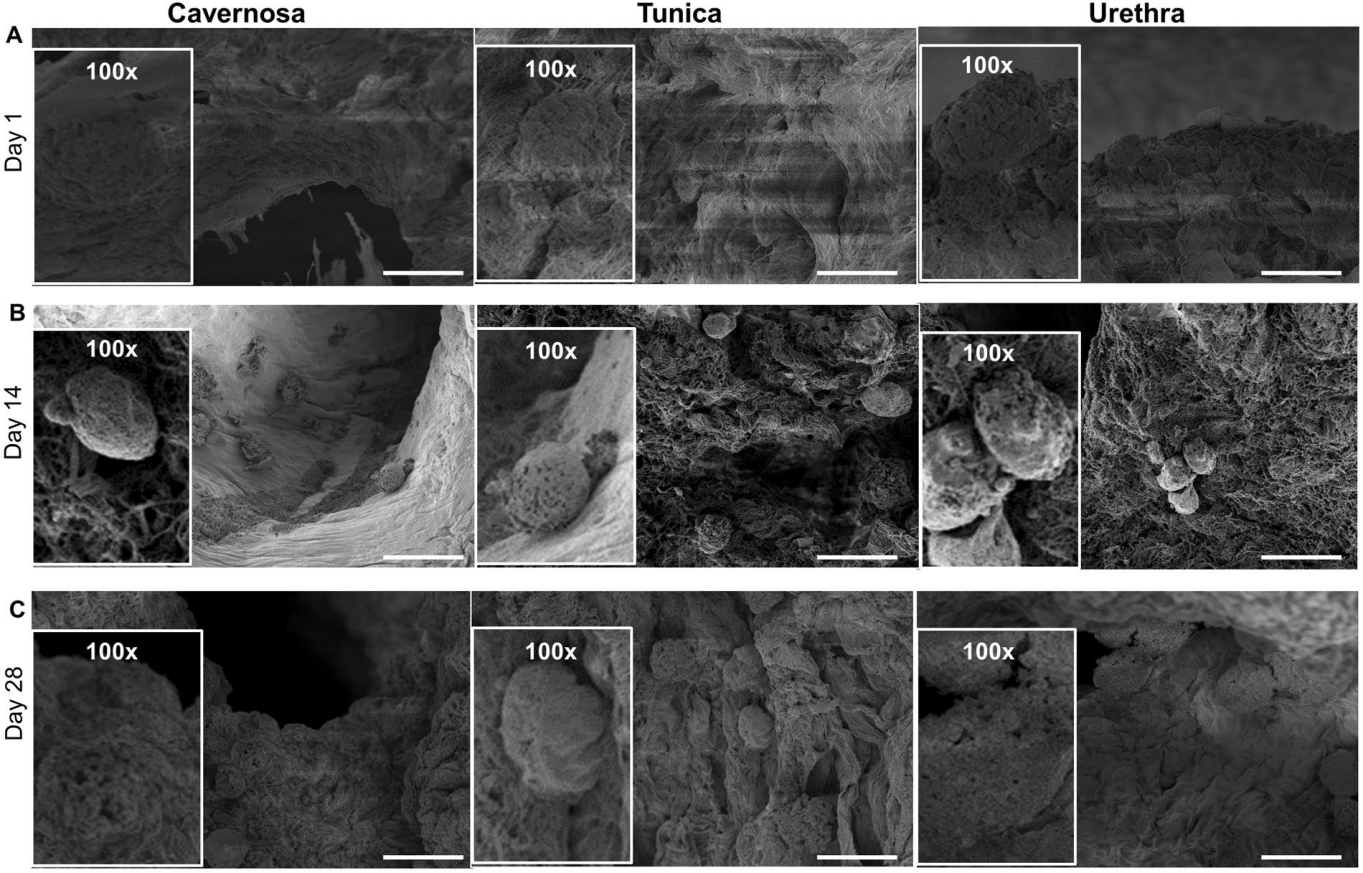
Scanning electron microscopy after scaffold reseeding with SVFs at day 4, day 14 and day 28. SEM images of three components of penis (cavernosa, tunica and urethra) at days 4 (**A**), 14 (**B**) and 28 (**C**) illustrating successful cell adhesion and proliferation, with the highest cell density seen in cavernosa. Scale bar: 20 µm.

The presence of cells was characterized using SEM (Fig. 7A), confirming cell retention and adhesion inside the three parts of scaffolds 4 days after penile scaffold repopulation. The increasing cell density post day 14 and day 28 (Fig. 7B,C) is consistent with the observation of Live/Dead staining, which confirms the proliferation of cells inside the decellularized penis scaffold. In particular, cell lining along the lumen structures in the cavernosa may indicate potential for the scaffold to guide functional behavior.

**Figure 8:**
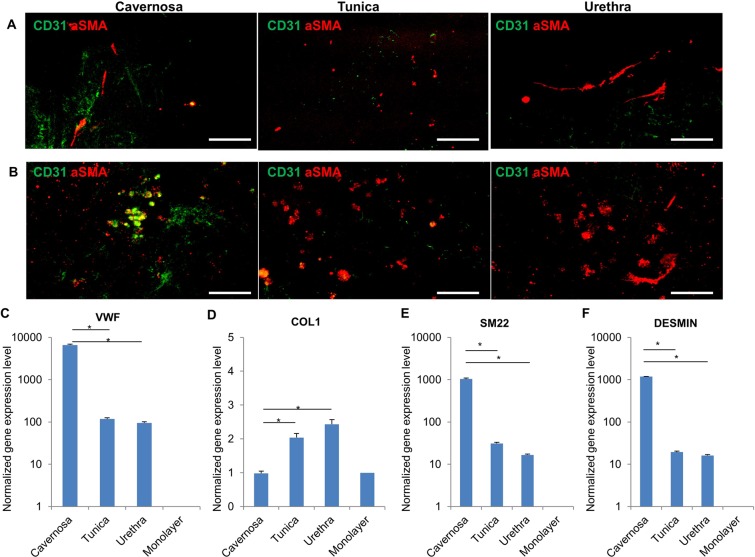
Cell Marker Expression after penile scaffold reseeding. Immunofluorescence of SVFs-seeded scaffold at days 14 (**A**) and 28 (**B**) for endothelial cell marker (CD31) and smooth muscle cell marker (aSMA) across the cavernosa, tunica and urethra. A greater degree of endothelial differentiation was seen in the cavernosa. (**C**–**F**) qRT-PCR analysis of VWF, COL1, SM22 and DESMIN respectively after SVFs seeding on decellularized penile scaffold (N = 3) at different anatomical regions (cavernosa, tunica and urethra) after 2 weeks. All gene expression levels are significantly higher in decellularized scaffold than mono-layer culture as control. (*P < *0*.*05)*. Scale bar: 100 µm.

Immunofluorescent staining was performed to detect the differentiation of cells. The confocal images (Fig. 8A) revealed some aSMA positive cells in the scaffold, indicating the existence of differentiated smooth muscle cells post cell seeding at day 14. Furthermore, at day 28 some CD31 positive cells were observed in the cavernosa part, which demonstrated the presence of differentiated endothelial cells from SVFs (Fig. 8B). The qPCR data (Fig. 8C–F) demonstrates that the SVFs-populated penis scaffold has significantly higher expression level of vWF, SM22 and desmin than 2D monolayer cell culture, indicating the potential capacity of decellularized scaffold for guiding cell differentiation towards major cell types of penis tissue (endothelial cells and smooth muscle cells). In addition, vWF, SM22 and desmin were significantly higher in expression in the cavernosa than tunica and urethra scaffolds, which is consistent with the immunofluorescent staining results. The tiled immunofluorescent image of the transverse section of the whole penis revealed that endothelial cells were abundantly distributed throughout the penis tissue, whereas smooth muscle cells were located mainly in cavernosa, less in urethra parts and no signal in the tunica (Supp Fig. 4). These data suggest that our perfusion-based decellularization protocol could yield an acellular penile scaffold which provides a suitable milieu for cell adhesion and differentiation to yield desired genitourinary behavior.

## Discussion

In this work we present a novel protocol for decellularizing a whole organ penile scaffold that reduces antigenic content to clinically acceptable levels while maintaining architectural integrity and biological properties. Such a scaffold could represent a new solution in cases of total penile loss after cancer or trauma or in transgender surgeries,  cases where the incidence is increasing rapidly.

The penis has three main sub-organs: two paired columns of corpora cavernosa that are involved in erectile function and the corpus spongiosum, which houses the urethra^21^. Each of the three is individually invested by a thick, fibrous tissue known as the tunica albuginea^22^. The combined structures are then covered by a thick fascial layer known as Buck’s fascia, followed by Dartos fascia^21,22^. The cellular heterogeneity and complexity of this system renders it a challenging target and it is unsurprising that most efforts to date have focused on only small subunits of the organ, e.g., the urethra or corpora.

The great majority of reports in the literature are on the use of rabbit models to study regeneration of the isolated corpora cavernosa. In 2003, the Atala group decellularized corporal slices with implantation into nude mice, with later reports enhancing reseeding methods^15^ and extending the work to successful restoration of full-length rather than segmental corporal defects in rabbits^14^. Song *et al*. showed that human umbilical smooth muscle cells could function similarly to rabbit corporal smooth muscle after reseeding. The Zhang group obtained muscle-derived stem cells from rabbit skeletal muscle and successfully used it for corporal reseeding^23^ while in a later report they used VEGF-overexpression to improve contractility of the resulting corpora^24^. Two recent reports have shown that bone marrow derived MSCs can be used for rabbit corporal tissue regeneration^20,25^.

Urethral decellularization for scaffold use has received far less attention. The Atala group used tubularized porcine bladder for urethral reconstruction in rabbits^26^. The first whole decellularization of a porcine urethra for rabbit implantation was recently reported in 2017 by Simoes *et al*.^16^. Unlike in corporal tissue engineering, a key challenge here is maintaining the patency of the urethral lumen, which our approach appears to have achieved.

Few studies exist in the literature with reports of successful human penile tissue decellularization. Egydio *et al*. in 2015 only decellularized small fragments (5 mm) of the distal glans skin rather than the functionally unique anatomical penile structures and has limited relevance to penile reconstruction^27^. In two separate projects, Kajbafzadeh *et al*. in 2017 isolated human cavernosa^28^ and urethra^29^ respectively for decellularization and successfully demonstrated small animal biocompatibility. The scaffold reported in our study is substantially more complex than any reported to date due to both (1) the anatomical complexity and increased scaffold diameter from using the whole organ with corpora and urethra rather than only one sub-unit and (2) the use of human penile specimens, with far greater scaffold size representing an additional obstacle but also an essential step to clinical translation. To surmount these challenges, we developed a novel hybrid decellularization approach combining vascular perfusion, urethral tube perfusion and standard external diffusion.

We have demonstrated the ability of the scaffold to support cell adhesion and ingrowth. However, large-scale reseeding of the entire scaffold will undoubtedly present an even greater challenge. We demonstrated post-decellularization patency of the cavernosal arteries, which our micro-CT data suggests offers the ability to access the microvascular network spanning most of the scaffold. Another limitation of this work is that, while adipose-derived cells were used here for proof-of-concept, differentiation prior to reseeding and multiple cell lineages will likely be required to restore the complex cellular morphology of the penis. Future work will report our approach via restoring the endothelium to vascular channels of the scaffold to facilitate further recellularization in a bench bioreactor setup, which has shown promising early results.

### Conclusion

In this work we have reported the first-ever protocol for processing whole-organ human penile specimens to create an off-the-shelf scaffold for total penile tissue engineering. The use of a hybrid decellularization scheme combining micro-arterial perfusion, urethral tube perfusion and standard external diffusion enabled the creation of a large scaffold with removal of immunogenic components, while maintaining an intact extracellular matrix structure and successfully supporting cell reseeding. Ultimately, we aim to fabricate a full scale, high-anatomic-fidelity human penile scaffold with the potential for more successfully restoring cosmetic, urinary and sexual function.

## Supplementary information

Supplementary Information

## Data Availability

The data that support the findings of this study are available from the corresponding author, DC, upon receipt of reasonable request.
