# Supplementary material for: Complete Human Penile Scaffold for Composite Tissue Engineering: Organ Decellularization and Characterization
Source: Sci Rep. 2019 Nov 8;9:16368. doi: 10.1038/s41598-019-51794-6 (PMC6841966; doi:10.1038/s41598-019-51794-6)
Supplement: Supplementary file 1 — Supplementary Information [file 41598_2019_51794_MOESM1_ESM.pdf]

Complete Human Penile Scaffold for Composite Tissue Engineering: Organ Decellularization and Characterization

Authors:
Yu Tan PhD[1,2]; Wilmina N. Landford, MD[1,2]; Matthew Garza, BA[1,2]; Allister Suarez, PhD[1,2]; Zhengbing, Zhou[3]; *Devin Coon MD, MSE[1,2]
[1] Department of Plastic & Reconstructive Surgery, Johns Hopkins University School of Medicine, 600 N Wolfe Street, Baltimore, Maryland, USA, 21287
[2] Translational Tissue Engineering Center, Department of Biomedical Engineering, Johns Hopkins University Whiting School of Engineering, Baltimore, Maryland, USA
[3] Department of Hand & Microsurgery, Xiangya Hospital of Central South University, 87 Xiangya Road, Changsha, Hunan Province, P. R. China, 410008

*Corresponding Author
Devin Coon MD, MSE
Department of Plastic & Reconstructive Surgery,
Johns Hopkins University School of Medicine
Translational Tissue Engineering Center, Department of Biomedical Engineering,
Johns Hopkins University Whiting School of Engineering
600 N Wolfe Street
Carnegie, 136
Baltimore, MD 21287

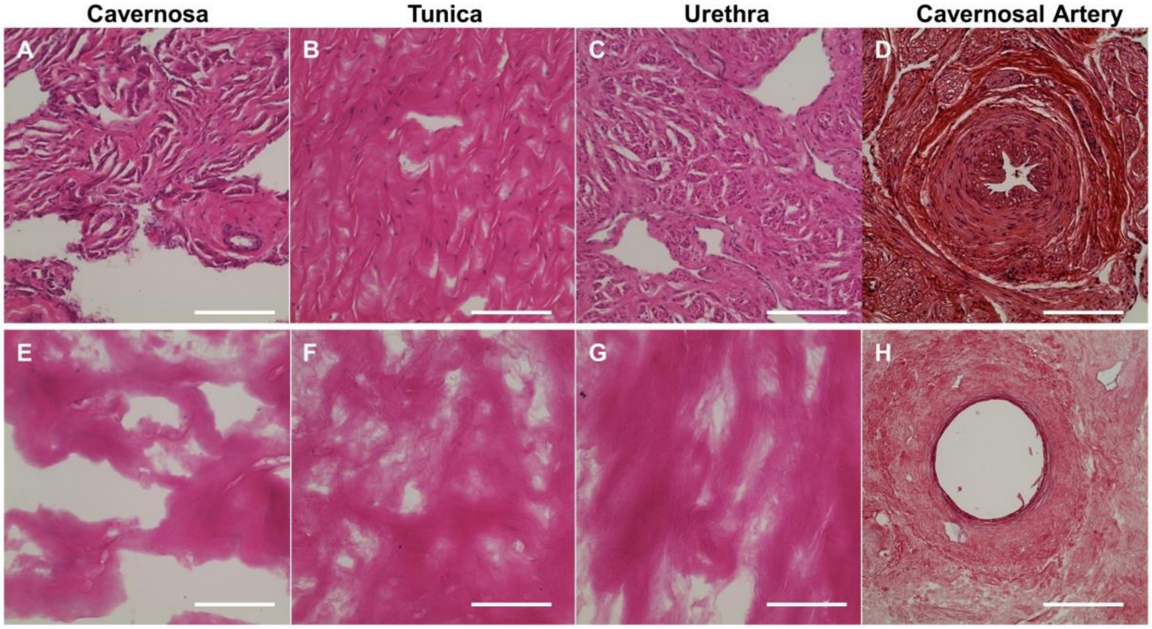

Supplementary Figure 1. H&E staining for characterization of decellularization across each anatomical unit. (A-D)
before and (E-H) after decellularization in cavernosa, tunica, urethra and cavernosal artery respectively. Scale bar:200μm.

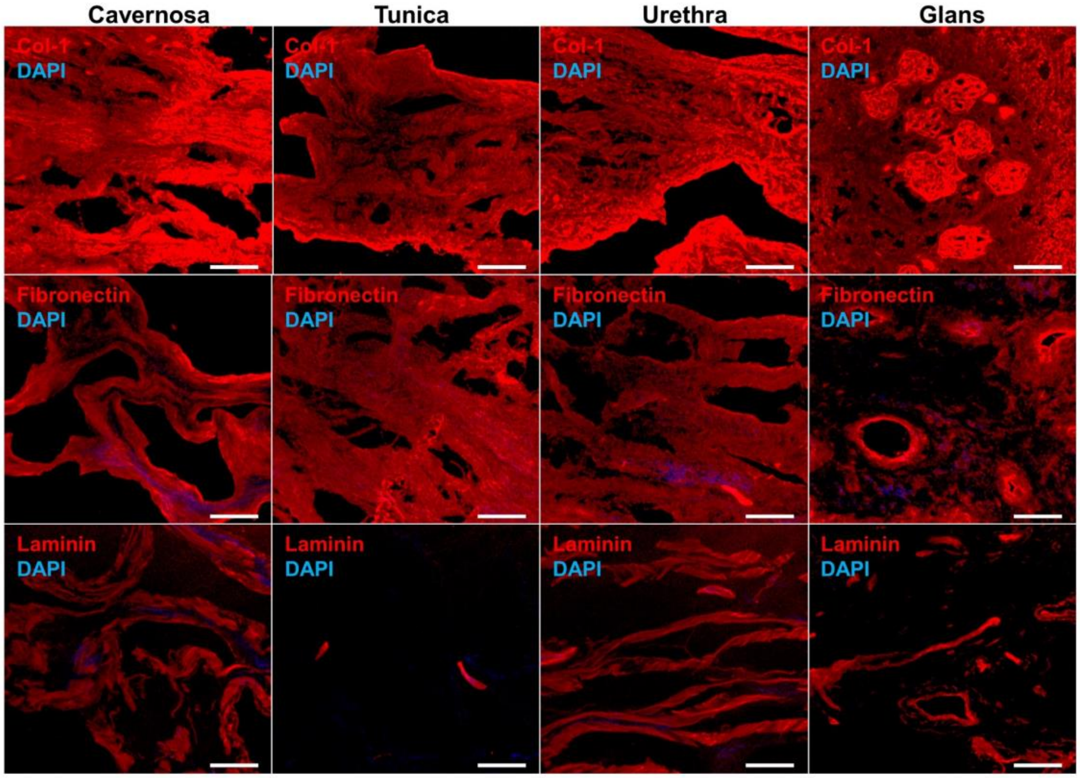

Supplementary Figure 2. Immunofluorescence staining of decellularized penile scaffold across each anatomical unit. Immunofluorescent staining of ECM proteins (Collagen-1, Fibronectin and Laminin) after decellularization in the cavernosa, tunica albuginea, urethra, and glans. Scale bar:100μm.

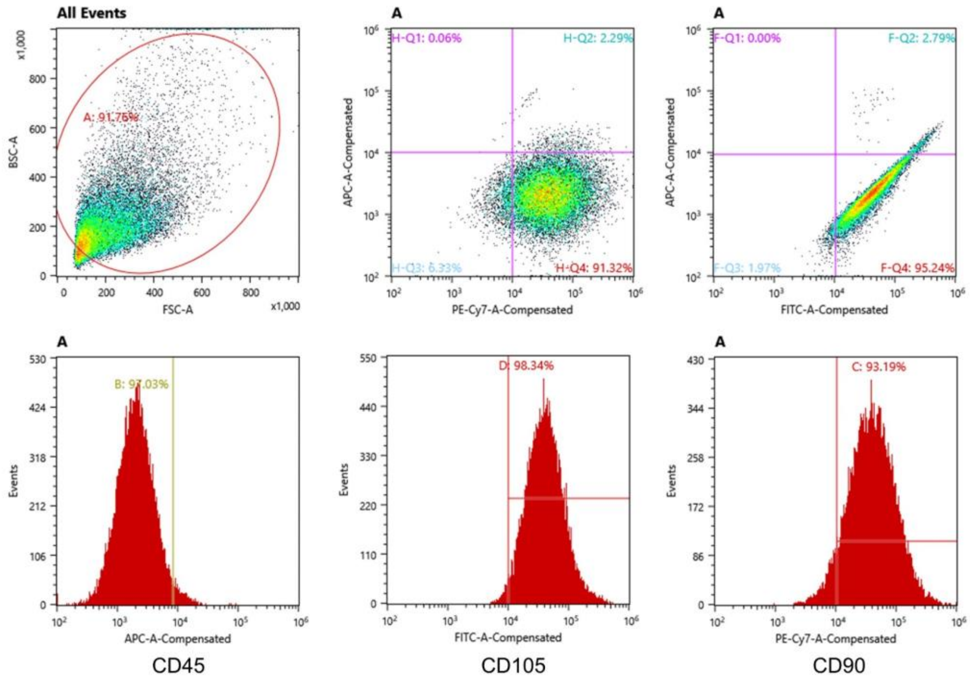

Supplementary Figure 3. Flow cytometry of ADSC cells used for scaffold reseeding.
Flow cytometry data showed that
SVFs obtained from human adipose tissue were negative for CD45 (97.03%) and positive for CD105 (98.34%) and CD90
(93.19%).

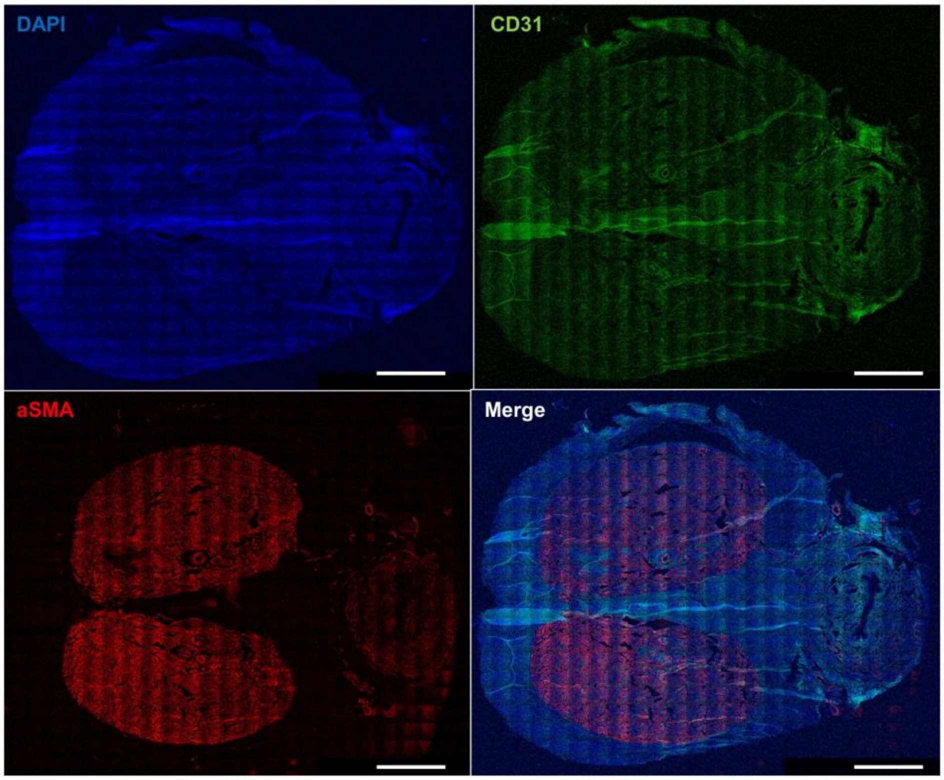

Supplementary Figure 4. Immunofluorescent staining of cross section of whole penis. Immunofluorescent staining of cross section of native penis tissue with endothelial cell marker (CD31) and smooth muscle cell marker (aSMA). Scale bar: 5mm.
